# *Caenorhabditis elegans* Protein Arginine Methyltransferase PRMT-5 Negatively Regulates DNA Damage-Induced Apoptosis

**DOI:** 10.1371/journal.pgen.1000514

**Published:** 2009-06-12

**Authors:** Mei Yang, Jianwei Sun, Xiaojuan Sun, Qinfang Shen, Zhiyang Gao, Chonglin Yang

**Affiliations:** 1 Key Laboratory of Molecular and Developmental Biology, Institute of Genetics and Developmental Biology, Chinese Academy of Sciences, Beijing, China; 2 Graduate School, Chinese Academy of Sciences, Beijing, China; Stanford University Medical Center, United States of America

## Abstract

Arginine methylation of histone and non-histone proteins is involved in transcription regulation and many other cellular processes. Nevertheless, whether such protein modification plays a regulatory role during apoptosis remains largely unknown. Here we report that the *Caenorhabditis elegans* homolog of mammalian type II arginine methyltransferase PRMT5 negatively regulates DNA damage-induced apoptosis. We show that inactivation of *C. elegans prmt-5* leads to excessive apoptosis in germline following ionizing irradiation, which is due to a CEP-1/p53–dependent up-regulation of the cell death initiator EGL-1. Moreover, we provide evidence that CBP-1, the worm ortholog of human p300/CBP, functions as a cofactor of CEP-1. PRMT-5 forms a complex with both CEP-1 and CBP-1 and can methylate the latter. Importantly, down-regulation of *cbp-1* significantly suppresses DNA damage-induced *egl-1* expression and apoptosis in *prmt-5* mutant worms. These findings suggest that PRMT-5 likely represses CEP-1 transcriptional activity through CBP-1, which represents a novel regulatory mechanism of p53-dependent apoptosis.

## Author Summary

Protein arginine methylation is an important posttranslational modification. Aberrant expression of protein arginine methyltransferases (PRMTs) are found in a wide variety of human diseases, especially in many cancers. Given that deregulation of apoptosis is usually related to tumorigenesis, it is not known whether PRMT–mediated protein arginine methylation plays a role in apoptosis. Here we employ the genetic tractable model organism *C. elegans* to explore the potential regulatory roles of PRMTs in apoptosis. We find that *C. elegans* PRMTs do not affect developmental cell deaths. However, genetic inactivation of the *C. elegans* homolog of the mammalian type II protein arginine methyltransferase PRMT5 causes excessive germ cell apoptosis in response to DNA damage. Our genetic analyses indicate that *prmt-5*–mediated apoptosis is dependent on the *C. elegans* p53 homolog CEP-1 and requires the core cell death pathway. We further demonstrate that loss of *prmt-5* leads to a specific up-regulation of the cell death initiator EGL-1 following DNA damage. Finally, we identify CBP-1, the *C. elegans* homolog of human p300/CBP, as a CEP-1 cofactor in *C. elegans*, and we provide genetic and biochemical evidence that PRMT-5 likely functions through CBP-1 to affect CEP-1/p53 transcriptional activity, thereby negatively regulating CEP-1/p53-dependent apoptosis.

## Introduction

Appropriate cellular response to DNA damage is critical for the maintenance of genome stability that is fundamental to the survival and development of organisms. In response to DNA damage, eukaryotic cells can activate checkpoint signaling pathways that are orchestrated by DNA damage sensors, mediators, transducers and effectors. In mammals, the damaged DNA is cooperatively recognized by the phosphoinositide 3-kinases ATM or ATR and the protein complexes Rad9-Rad1-Hus1 (9-1-1) and RFC-Rad17 [1]. ATM and ATR initiate a phosphorylation cascade that eventually leads to the stabilization of the tumor suppressor p53. By selectively activating cell cycle controlling genes such as p21 and proapoptotic genes such as Bax, Puma and Noxa, p53 can induce either cell cycle arrest that allows the repair of damaged DNA, or apoptosis that eliminates those over-damaged cells in which DNA lesions are irreparable [1],[2]. In addition to phosphorylation, the activation of p53 also involves other protein modifications including methylation and acetylation that are implicated in increasing p53 protein stability [3]. Moreover, the activity of p53 is also regulated by transcription coactivators such as histone acetyltransferases p300 and CBP which are recruited by p53 to form transcription initiation complex to facilitate the transcription of p53 target genes [4]–[6].

DNA damage sensing and signaling pathways are evolutionarily conserved across diverse species ranging from *C. elegans* to humans. In *C. elegans*, the p53 homolog CEP-1 acts as a key effector to mediate germ cell apoptosis triggered by ionizing irradiation [7],[8]. Following DNA damage, CEP-1/p53 transcriptionally activates the cell death initiator EGL-1, a *C. elegans* BH3-only protein analogous to the mammalian p53 targets Puma and Noxa, leading to the activation of the core cell death pathway that is essentially controlled by several evolutionarily conserved apoptotic factors, including the Bcl-2-like antiapoptotic protein CED-9, the *C. elegans* Apaf-1 homolog CED-4, and the caspase CED-3 [9],[10]. In addition, inactivation of components in the checkpoint signaling pathways also gives rise to abnormal apoptosis of germ cells following DNA damage. For example, mutations in *mrt-2*, *hus-1* and *clk-2*, which encode the *C. elegans* homologs of mammalian Rad1, Hus1 and Rad5, respectively, suppress cell cycle arrest and germ cell apoptosis induced by γ-irradiation [9],[11],[12]. Similarly, inactivation of *atm-1* and *atl-1*, the *C. elegans* homologs of mammalian ATM and ATR, respectively, suppresses both DNA damage-induced cell cycle arrest and apoptosis in *C. elegans* germline [13]. These facts indicate that the DNA damage signaling pathway leading to apoptosis in *C. elegans* is essentially similar to that in mammals, which makes *C. elegans* an excellent model organism for identifying novel components involved in cellular response to DNA damage.

Protein arginine methyltransferases (PRMTs) are a family of proteins that catalyze the addition of one or two methyl groups to the guanidine nitrogen atoms of arginine, a process of posttranslational modification termed protein arginine methylation [14],[15]. PRMTs have been found in diverse species and 11 members are identified in mammals [14],[15]. Depending on the methylated forms of arginine residues of their substrates, mammalian PRMTs are classified into two types. The type I PRMTs, including PRMT1, 3, 4, 6 and 8, catalyze asymmetric dimethylation of arginine residues (aDMA). In comparison, the type II PRMTs, including PRMT5, 7 and 9, catalyze symmetric dimethylation of arginine residues (sDMA) [15]. In recent years, a growing body of evidence indicates that protein arginine methylation plays important roles in regulating multiple cellular processes such as transcriptional regulation, signal transduction, DNA repair as well as RNA processing [14]. The regulation of transcription by PRMTs generally involves the recruitment of these proteins to promoters by transcription factors and methylation of histone tails [14], or methylation of transcription coactivators such as p300/CBP [16],[17] and transcription elongation factors such as SPT5 [18]. Recent studies have also shown that protein arginine methylation is likely involved in cellular response to DNA damage. For example, it has been reported that PRMT1, CARM1/PRMT4 and p300 function cooperatively to promote p53 transcriptional activity on the cell cycle controlling gene GADD45 [19]. In addition, it was found that PRMT2 promotes apoptosis by negatively regulating NF-κB independently of its methylation activity, which is also implicated in DNA damage-induced apoptosis [20]. Nevertheless, whether other PRMTs are involved in cellular response to DNA damage, especially in p53-dependent apoptosis, still remains largely unknown. Moreover, aberrant expression of PRMTs are found to associate with a wide variety of human diseases including many cancers, but the underlying mechanisms still need to be further elucidated [14].

To better understand the signaling mechanisms underlying cellular response to DNA damage, we took advantage of the genetic tractable model organism *C. elegans* to determine whether PRMTs and protein arginine methylation are involved in p53-dependent apoptosis. We found that inactivation of *prmt-5*, which encodes the *C. elegans* homolog of mammalian PRMT5, significantly increased germ cell apoptosis following ionizing radiation. Our genetic analyses indicate that *prmt-5*-mediated apoptosis is dependent on *cep-1*/p53 and requires the core cell death pathway. Furthermore, we provide evidence that CBP-1, the *C. elegans* homolog of mammalian p300/CBP, acts as a transcription coactivator of CEP-1 and can be methylated by PRMT-5. The formation of a tripartite complex among PRMT-5, CEP-1 and CBP-1 and the methylation of CBP-1 by PRMT-5 likely repress the transcriptional activation of the cell death initiator EGL-1 in response to DNA damage. Our findings not only demonstrate that PRMT-5 is a novel component critical for DNA damage-induced apoptosis in *C. elegans*, but also suggest a negative regulatory mechanism underlying p53-dependent apoptosis.

## Results

### Inactivation of *C. elegans prmt-5* Leads to Excessive Germ Cell Apoptosis following DNA Damage

To identify putative protein arginine methyltransferases in *C. elegans*, we used the sequences of individual mammalian PRMTs to search the *C. elegans* genome database. 6 putative open reading frames either containing conserved motifs of arginine methyltransferase or sharing other homology with mammalian PRMTs were obtained. Based on their sequence similarity to mammalian PRMTs, we designated these genes *prmt-1*, *-2*, *-3*, *-4*, *-5*, and *-6*, respectively (*prmt* represents protein arginine methyl transferase) (Figure S1A, S1B). Next, we examined whether these *prmt* genes are involved in *C. elegans* programmed cell death using RNA interference (RNAi) to knock down their expressions. Our time-course analyses of both embryonic and germ cell corpses indicated that inactivation of these genes did not obviously affect the cell death profiles, which suggests that protein arginine methylation may not be involved in developmental cell deaths in *C. elegans* (data not shown). However, when irradiated with γ-ray, animals pre-treated with *prmt-5* RNAi showed an obvious increase of germ cell corpses compared with control RNAi-treated worms, suggesting that *prmt-5* is likely involved in DNA damage-induced apoptosis (Figure S1C).

**Figure 1:**
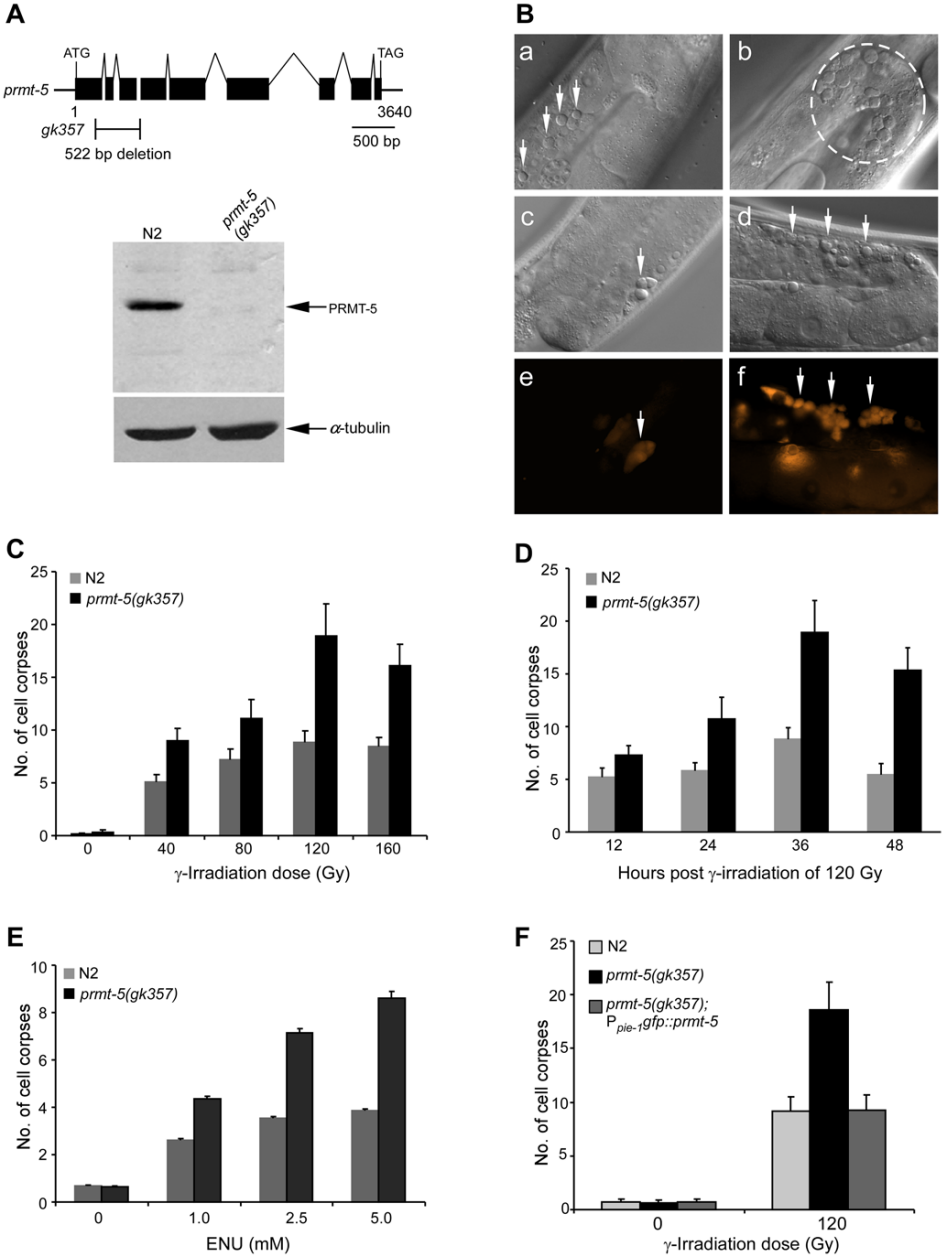
*prmt-5* mutation causes excessive germ cell apoptosis in response to DNA damage. (A) *prmt-5(gk357)* is likely a null allele. The top panel shows the schematic representation of *prmt-5(gk357)* deletion mutation. Solid boxes indicate exons and waved lines indicate introns. The fragment below the gene indicates the deletion region and size. The bottom panel shows the endogenous expression of PRMT-5 in N2 and *prmt-5(gk357)* animals detected by using anti-PRMT-5 antibody. α-tubulin indicates the loading control of the samples. (B) γ-irradiation induces excessive germ cell apoptosis in *prmt-5(gk357)* animals. Young adult worms were treated with γ-irradiation of 120 Gy. 36 h later, germ cell corpses were examined under DIC optics and representative images of cell corpses are shown for N2 (a) and *prmt-5(gk357)* animals (b), respectively. Irradiated worms were also stained with acridine orange (AO) and both DIC and AO-positive germ cell corpses were shown for N2 (c, e) and *prmt-5(gk357)* mutants (d, f). Germ cell corpses are indicated by arrows (a, c–f) or fragmented circle (b). (C, D) Quantitative analysis of germ cell apoptosis induced by γ-irradiation. Germ cell corpses from one gonad arm of each animal were scored 36 h post irradiation of indicated doses (C), or at indicated time points post irradiation of 120 Gy (D). At least 20 worms were scored at each radiation dose or time point. Error bars represent standard error of the mean (SEM). (E) ENU induces excessive germ cell apoptosis in *prmt-5(gk357)* mutants. Young adult worms were treated with ENU at indicated concentrations for 4 h and germ cell corpses from one gonad arm of each animal were scored 24 h post ENU treatment. At least 20 worms were scored at each concentration point. (F) Rescuing activity of P*_pie-1_gfp::prmt-5* in *prmt-5(gk357)* germline. Worms were irradiated with γ-ray of 120 Gy and germ cell corpses were scored as above 36 h after irradiation.

*C. elegans prmt-5* gene is defined by the open reading frame C34E10.5 located on the linkage group III, which encodes a protein of 734 amino acids. The predicted worm PRMT-5 protein shows the highest sequence similarity to human type II protein arginine methyltransferase PRMT5 (34% sequence identity and 48% similarity, respectively). The sequence similarity is particularly strong between the residues 105 to 730 of *C. elegans* PRMT-5 and residues 58 to 633 of human PRMT5. *C. elegans* PRMT-5 also shares homology with yeast Skb1 and Drosophila Dart1 (Figure S2). Previously, a genome-wide RNAi screen showed that inactivation of *prmt-5*/C34E10.5 could cause increased level of spontaneous mutation in *C. elegans*, suggesting that *prmt-5* is important for genome stability [21]. However, it is not known whether *prmt-5* also plays a role in DNA damage-induced apoptosis. To further determine this, we analyzed a mutant strain *prmt-5(gk357)* containing a deletion of 522 bp that removes a small region of the exon 1 and the whole exons 2 and 3 of *prmt-5* genomic locus (Figure 1A). Using an antibody generated against recombinant PRMT-5, we detected the expression of PRMT-5 in wild type but not in *prmt-5(gk357)* mutants, indicating that *prmt-5(gk357)* is likely a strong loss-of-function allele (Figure 1A). *prmt-5(gk357)* animals display no obvious developmental defects except that the growth rate is slightly lower than that of wild-type animals. Similar to *prmt-5(RNAi)* worms, *prmt-5(gk357)* animals do not show discernible defects in developmental cell deaths (data not shown). However, when exposed to γ-irradiation (IR), *prmt-5(gk357)* mutants exhibited a strong increase of germ cell apoptosis compared with that in wild-type animals. The IR-induced apoptosis in *prmt-5(gk357)* animals occurred mostly in the germline meiotic region containing pachytene-stage cells and the apoptotic cells displayed disc-like structures which were morphologically indistinguishable from those in wild-type worms (Figure 1B(a–b)). A further staining of irradiated animals with acridine orange (AO), a fluorescence dye that preferentially stains cell corpses internalized in engulfing cells, also indicated that *prmt-5(gk357)* worms contained significantly more AO-positive germ cell corpses than wild-type animals (Figure 1B(c–f)). Collectively, these data indicate that *prmt-5* loss-of-function mutation leads to excessive germ cell apoptosis following γ-irradiation.

We evaluated the dosage effect of IR on germ cell apoptosis in *prmt-5(gk357)* animals by exposing them to different doses of γ-irradiation. In both wild-type and *prmt-5(gk357)* animals, IR induced an increase of germ cell apoptosis in a dose-dependent manner, but the number of germ cell corpses in *prmt-5(gk357)* worms was significantly higher than in wild-type animals at all tested irradiation doses (Figure 1C). The appearance of germ cell corpses in *prmt-5(gk357)* mutants reached a peak 36 h post irradiation of 120 Gy, which was about 2 times of that in wild-type animals (Figure 1C and 1D). To determine whether other DNA-damage agents can also induce excessive germ cell apoptosis in *prmt-5(gk357)* mutants, we treated *prmt-5(gk357)* animals with ethylnitrosourea (ENU), a DNA–alkylating agent that can cause a broad spectrum of DNA lesions. Our results indicate that ENU induced elevated germline apoptosis in both wild-type and *prmt-5(gk357)* animals in a concentration-dependent manner. Moreover, significantly more germ cell corpses were observed in the *prmt-5(gk357)* mutants than in wild-type animals at all tested ENU concentrations (Figure 1E). Importantly, the excessive germ cell apoptosis observed in the *prmt-5(gk357)* mutants following γ-irradiation was strongly reduced when a GFP::PRMT-5 fusion protein was overexpressed under the control of the *pie-1* promoter (P*_pie-1_*) which specifically drives gene expression in germ cells [22], indicating that germline-specific expression of PRMT-5 rescued the germ cell apoptosis phenotype in the *prmt-5(gk357)* mutants (Figure 1F). Taken together, these findings suggest that *prmt-5* likely antagonizes DNA damage-induced apoptosis in *C. elegans*.

### *prmt-5* Acts through the Core Cell Death Pathway and Requires Checkpoint Signaling

**Figure 2:**
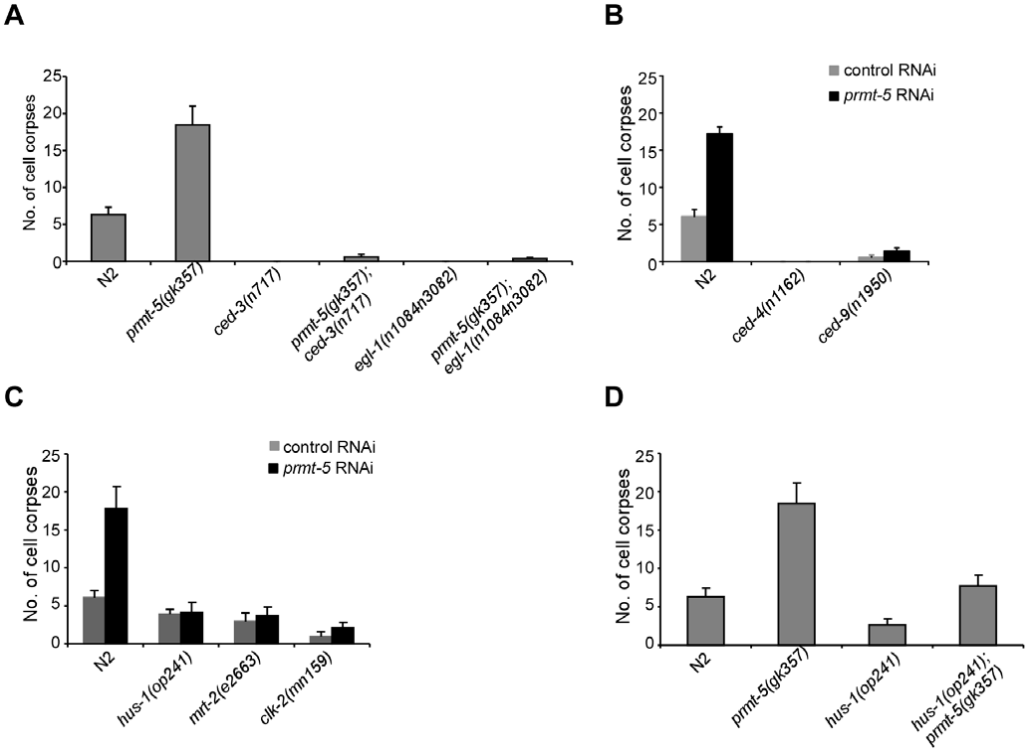
Epitasis analysis of *prmt-5*-mediated apoptosis. (A) Quantification of IR-induced germ cell apoptosis in N2, *prmt-5(gk357)*, *ced-3(n717)*, *egl-1(n1084 n3082)* single mutants and *prmt-5(gk357);ced-3(n717)* as well as *prmt-5(gk357);egl-1(n1084 n3082)* double mutants. Young adult animals were irradiated with γ-ray of 120 Gy and analyzed 36 h post irradiation. Germ cell corpses from one gonad arm of each animal were scored for at least 20 animals. Error bars represent SEM. (B) Quantification of germ cell apoptosis in control RNAi- and *prmt-5* RNAi-treated N2, *ced-4(n1162)* and *ced-9(n1950gf)* mutants following γ-irradiation. Worms were irradiated and germ cell corpses were scored and analyzed as in (A). (C) Quantification of IR-induced germ cell apoptosis in control RNAi- and *prmt-5* RNAi-treated N2, *hus-1(op241)*, *mrt-2(e2663)* and *clk-2(mn159)* animals. Worm treatment and germ cell corpse analysis were performed as in (A). (D) Quantification of IR-induced germ cell apoptosis in N2, *prmt-5(gk357)*, *hus-1(op241)* single mutants and *hus-1(op241);prmt-5(gk357)* double mutants. Worm treatment and germ cell corpse analysis were performed as in (A).

Several lines of evidence have shown that germ cell apoptosis induced by DNA damage requires the core cell death pathway because mutations of genes essential for programmed cell death, including *ced-3*, *ced-4*, *ced-9* and *egl-1*, block such cell death. To determine whether the strong increase of IR-induced germ cell apoptosis in *prmt-5(gk357)* mutants is dependent on the core cell death pathway, we generated *prmt-5(gk357);ced-3(n717)* and *prmt-5(gk357);egl-1(n1084 n3082)* double mutants and found that germ cell apoptosis was barely induced by IR in these worms (Figure 2A). Moreover, in *prmt-5* RNAi-treated *ced-4(n1162)* loss-of-function and *ced-9(n1950)* gain-of-function mutants, IR-induced germ cell apoptosis was either abrogated or strongly suppressed as compared with that in *prmt-5* RNAi-treated wild-type animals (Figure 2B). These results suggest that *prmt-5* acts through the core cell death pathway to regulate DNA damage-induced apoptosis.

It was reported previously that mutations in *mrt-2*, *hus-1* and *clk-2*, which encode *C. elegans* homologs of mammalian checkpoint signaling components Rad1, Hus1 and Rad5, respectively, inhibit both DNA damage-induced cell cycle arrest and apoptosis in *C. elegans* [9],[11],[12]. The progeny of checkpoint mutants are also hypersensitive to IR treatment owing to defects in DNA repair [12],[23]. Although *prmt-5(gk357)* animals laid fewer eggs than wild-type worms after irradiation, which was potentially resulted from excessive germline apoptosis, the survival of *prmt-5(gk357)* progeny was comparable to that of wild-type animals (Text S1, Table S1). In addition, *prmt-5(gk357)* worms displayed similar cell cycle arrest in germline mitotic region to that in wild type following IR treatment (data not shown). Together, these data suggest that *prmt-5* does not act as a checkpoint gene to affect cell cycle progression; instead, its effect is likely specific to apoptosis in response to DNA damage. We thus asked whether checkpoint signaling affects *prmt-5*-mediated apoptosis upon DNA damage. To test that, we used RNAi to inactivate *prmt-5* in *hus-1(op241)*, *mrt-2(e2663)* and *clk-2(mn159)* mutants and induced germ cell apoptosis with γ- irradiation of 120 Gy. Our data indicate that mutations in *hus-1*, *mrt-2* and *clk-2* significantly inhibited IR-induced germline apoptosis in *prmt-5(RNAi)* worms (Figure 2C). Furthermore, the number of germ cell corpses was strongly reduced, but not entirely suppressed, in *hus-1(op241);prmt-5(gk357)* double mutants compared with that in *prmt-5(gk357)* single mutants after IR treatment (Figure 2D). These findings suggest that checkpoint signaling is important for *prmt-5*-mediated apoptosis, and *prmt-5* likely acts in parallel to, or downstream of, checkpoint genes to regulate apoptosis in response to DNA damage.

### *prmt-5(gk357)* Affects CEP-1/p53 Transcriptional Activity

**Figure 3:**
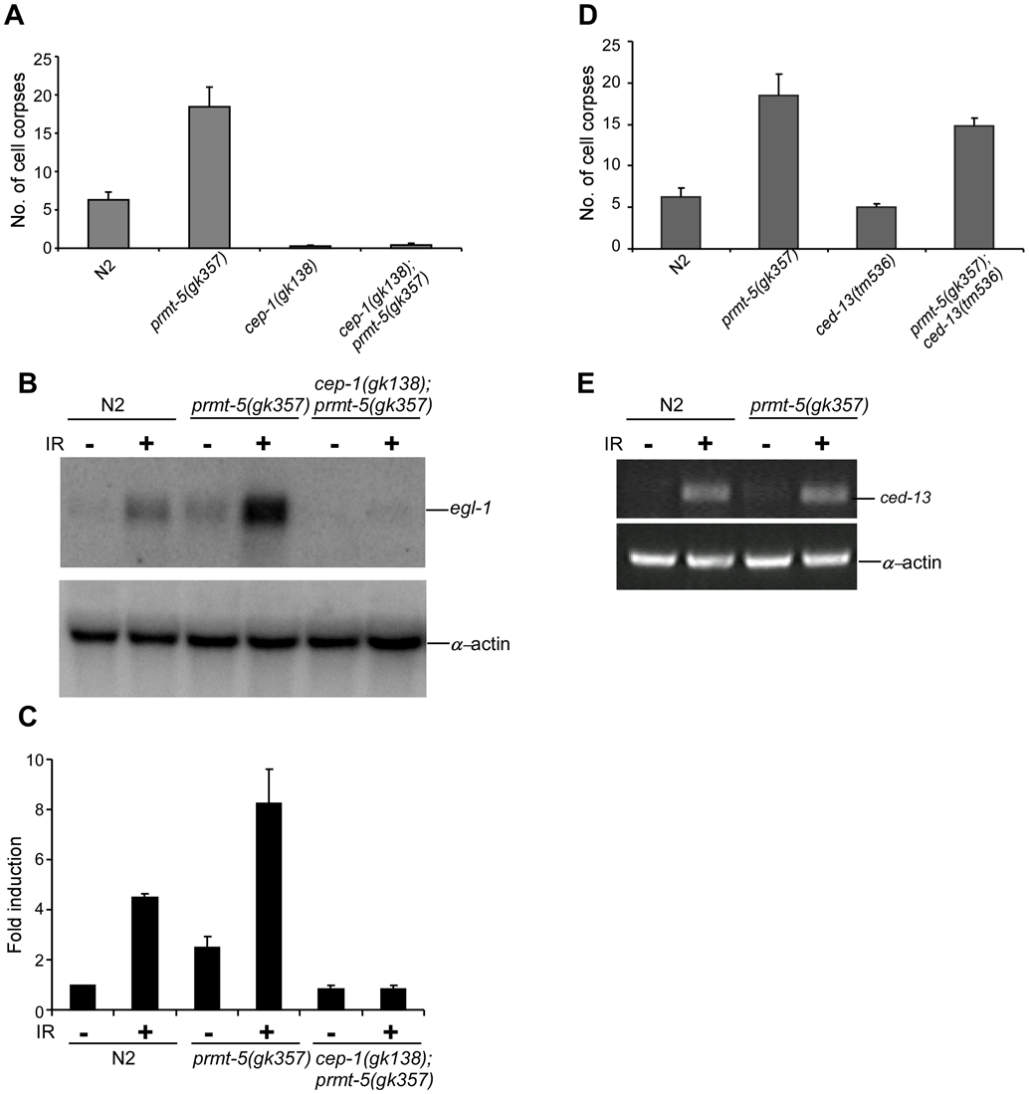
*prmt-5(gk357)* affects CEP-1 transcriptional activity in response to DNA damage. (A) Quantification of IR-induced germ cell apoptosis in N2, *prmt-5(gk357)*, *cep-1(gk138)* single mutants and *cep-1(gk138);prmt-5(gk357)* double mutants. Young adult animals were irradiated with γ-ray of 120 Gy and analyzed 36 h post irradiation. Germ cell corpses from one gonad arm of each animal were scored for at least 20 animals. Error bars represent SEM. (B) Northern blot analysis of *egl-1* mRNA in N2, *prmt-5(gk357)* and *cep-1(gk138);prmt-5(gk357)* worms treated with γ-irradiation. Young adult worms were treated with γ-irradiation of 120 Gy and total RNA were prepared 24 h post irradiation. *egl-1* expression was detected by using *egl-1* cDNA as probe. α-actin mRNA was probed as loading control of samples. Three independent Northern blot analyses were performed and the representative images are shown. (C) Relative fold induction of *egl-1* mRNA in indicated animals 24 h after γ-irradiation of 120 Gy. *egl-1* fold induction was averaged from three independent Northern blot analyses, which was quantified by using the software ImageQuant 5.2 and normalized with α-actin mRNA. (D) Quantification of IR-induced germ cell apoptosis in N2, *prmt-5(gk357)*, *ced-13(tm536)* single mutants and *prmt-5(gk357);ced-13(tm536)*double mutants. Worm treatment and germ cell corpse analysis were performed as in (A). (E) *ced-13* expression in N2 and *prmt-5(gk357)* mutants after γ-irradiation. Worms were treated as in (B) and *ced-13* expression was detected by using semi-quantitative RT-PCR; α-actin mRNA was used as internal control of PCR.

Previous studies have shown that CEP-1 transcriptionally activates *egl-1* in response to DNA damage [9],[10],[24]. Because *egl-1* loss of function blocked IR-induced apoptosis in *prmt-5(gk357)* animals (Figure 2A), we asked further whether CEP-1 activity is required for IR-induced excessive apoptosis in *prmt-5(gk357)* worms. To answer this, we constructed double mutants of *prmt-5(gk357)* with the *cep-1* deletion allele *gk138*. In *cep-1(gk138);prmt-5(gk357)* double mutants, no germ cell apoptosis was observed after γ-irradiation of 120 Gy (Figure 3A), suggesting that *prmt-5* functions upstream or at the level of *cep-1*. Further analyses did not reveal any obvious changes in mRNA or protein levels of *cep-1* in *prmt-5(gk357)* animals after irradiation (data not shown), indicating that *prmt-5* mutation likely does not affect either the transcription or mRNA stability or protein stability of *cep-1*.

To understand how *cep-1* and *egl-1* might be involved in IR-induced excessive apoptosis observed in *prmt-5(gk357)* animals, we examined whether the mRNA level of *egl-1* was affected in *prmt-5(gk357)* animals after being exposed to γ-irradiation. Using Northern blot analysis, we found that IR-induced *egl-1* mRNA level was significantly enhanced in *prmt-5(gk357)* mutants compared with that in wild type (Figure 3B). For example, in three independent Northern blot analyses, the average *egl-1* mRNA level was induced by about 4-fold in wild-type animals 24 h after irradiation (Figure 3C). In *prmt-5(gk357)* animals, the basal level of *egl-1* mRNA appeared slightly higher than in wild-type worms though it might not be sufficient for triggering excessive apoptosis under physiological condition, and *egl-1* expression was further increased by about 8-fold after irradiation as compared with that in non-irradiated wild-type animals (Figure 3B and 3C). When comparison was made between IR-treated wild-type and *prmt-5(gk357)* animals, we constantly observed a further increase of *egl-1* mRNA by 1.5- to 2.5-fold in *prmt-5(gk357)* mutants (Figure 3B and 3C). In contrast, in *cep-1(gk138);prmt-5(gk357)* double mutants, IR-induced *egl-1* expression was completely abrogated (Figure 3B and 3C), indicating that *cep-1* activity is absolutely required for IR-induced over up-regulation of *egl-1* mRNA caused by loss of *prmt-5* function.

*ced-13*, which encodes another BH3-only protein in *C. elegans*, was also reported to be transcriptionally activated by CEP-1 following DNA damage [25]. However, we found that *ced-13(tm536)* deletion, which likely represents a strong loss-of-function mutation of *ced-13* [25], did not suppress the IR-induced excessive germ cell apoptosis in *prmt-5(gk357)* animals (Figure 3D). By Northern blot analysis, we were not able to detect obvious expression of *ced-13* following γ-irradiation in either wild type or *prmt-5(gk357)* animals, which is likely due to a very low expression level of *ced-13*. Thus we used the more sensitive semi-quantitative RT-PCR assay to examine *ced-13* expression in response to DNA damage. As reported before [25], our results indicate that *ced-13* expression was increased in both wild type and *prmt-5(gk357)* animals after γ-irradiation. However, *ced-13* mRNA level was not significantly increased in *prmt-5(gk357)* mutants compared with that in wild-type animals (Figure 3E), which is consistent with that *ced-13(tm536)* deletion did not suppress IR-induced excessive germ cell apoptosis in *prmt-5(gk357)* animals (Figure 3D). In addition, our Northern blot analysis showed that the transcripts of *ced-3*, *ced-4* and *ced-9* were not changed in *prmt-5(gk357)* mutants after DNA damage (data not shown). Taken together, these findings suggest that *prmt-5* mutation results in a specific over up-regulation of *egl-1* which leads to excessive germ cell apoptosis following DNA damage.

### PRMT-5 Interacts with CEP-1

Since our genetic data indicate that *cep-1* is required for the IR-induced over up-regulation of *egl-1* and the excessive germ cell apoptosis caused by loss of *prmt-5* function, we next investigated how PRMT-5 may regulate CEP-1 transcriptional activity. In mammals, PRMT5 was found to regulate transcription by methylating symmetrically the arginine 3 residue of histone H4 (H4R3) and the arginine 8 residue of histone H3 (H3R8) [26]. We wondered whether *C. elegans* PRMT-5 could also methylate histone H4 or H3. Thus we prepared recombinant PRMT-5 and incubated it with core histones and the donor of methyl group, ^3^H-S-AdoMet (^3^H-SAM). Our results indicate that PRMT-5 methylated histone H4 but not H3 *in vitro* (Figure S3A), suggesting that histone H3 is less likely a substrate of PRMT-5 in *C. elegans*. We therefore examined whether PRMT-5 is important for H4R3 symmetric dimethylation (H4R3sMe2) *in vivo* as is mammalian PRMT5. Using an antibody that specifically recognizes H4R3sMe2, however, we did not detect significant difference in H4R3 symmetric dimethylation in germline between wild-type and *prmt-5(gk357)* animals (Figure S3B). Thus, worm PRMT-5 seems not to control the general status of H4R3sMe2 to regulate CEP-1 transcriptional activity.

**Figure 4:**
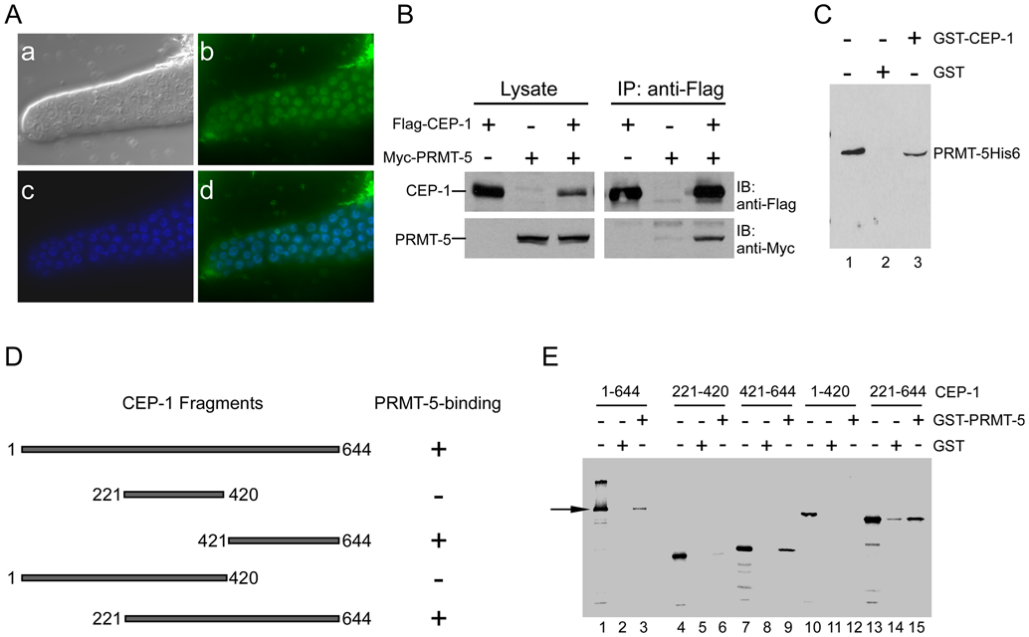
PRMT-5 interacts with CEP-1. (A) GFP::PRMT-5 localizes to nucleus in germline. The GFP::PRMT-5 fusion protein was expressed in germ cells under the control of the *pie-1* promoter (P*_pie-1_gfp::prmt-5*). Images of DIC (a), GFP (b), Hoechst 33342 staining (c) and the merged picture of GFP and Hoechst 33342 staining (d) are shown. (B) PRMT-5 and CEP-1 interact with each other in mammalian cells. Flag-CEP-1 and Myc-PRMT-5 were co-expressed in HEK293 cells and immunoprecipitated (IP) by using anti-Flag antibody. The precipitated proteins were detected by immuno-blotting (IB) with indicated antibodies. (C) PRMT-5 and CEP-1 directly interact *in vitro*. Purified PRMT-5His6 was incubated with GST-CEP-1 immobilized on glutathione sepharose beads for more than 2 h. After extensive wash, the bound PRMT-5His6 was detected by immuno-blotting with anti-PRMT-5 antibody. Lane 1 indicates 10% of input PRMT-5His6 protein. (D, E) PRMT-5 binds to the C-terminal region of CEP-1. The full-length or truncated CEP-1 proteins as indicated in (D) were *in vitro* translated and labeled with ^35^S and incubated with GST or GST-PRMT-5 immobilized on glutathione sepharose beads for more than 2 h. After extensive wash, the bound proteins were resolved by SDS-PAGE and detected by autoradiography (E). “+” indicates the existence of interaction between CEP-1 and PRMT-5 and “−” indicates no interaction in (D). In lanes l, 4, 7, 10 and 13 in (E), 10% of the *in vitro* translated proteins used for interaction were loaded. Arrow indicates the full-length CEP-1.

We then determined whether PRMT-5 was able to enter nucleus by examining the subcellular localization of a translational fusion protein GFP::PRMT-5 driven by the promoter of the germline-specific gene *pie-1* (P*_pie-1_gfp::rmt-5*). About 20% worms from an integrated transgenic line carrying P*_pie-1_gfp::prmt-5* displayed GFP signals, which were enriched in germline nuclei, suggesting that GFP::PRMT-5 was able to localize to nucleus (Figure 4A). Therefore we tested whether PRMT-5 could interact with CEP-1 by co-expressing Flag-tagged CEP-1 and Myc-tagged PRMT-5 in HEK293 cells and performing immunoprecipitation. Our results indicate that Myc-PRMT-5 was co-immunoprecipitated with Flag-CEP-1 (Figure 4B), suggesting that these two proteins can interact in mammalian cells. Furthermore, we used *in vitro* GST pull-down assay to determine if they could directly interact with one another. We found that the GST-CEP-1 fusion protein immobilized on glutathione sepharose beads, but not GST, interacted with purified PRMT-5His6 protein (Figure 4C). Reciprocally, GST-PRMT-5 interacted with the full-length CEP-1 (Figure 4D and 4E). Furthermore, ^35^S-labeled CEP-1(421–644), but not CEP-1(1–420) or CEP-1(221–420), interacted with GST-PRMT-5, indicating that the C-terminal region of CEP-1 was necessary and sufficient for its binding to PRMT-5 (Figure 4D and 4E). These data indicate that PRMT-5 and CEP-1 can directly and specifically interact with each other.

The direct interaction between PRMT-5 and CEP-1 promoted us to examine whether PRMT-5 could methylate CEP-1. *In vitro*, when incubated with the full-length GST-CEP-1 fusion protein or various truncations of GST-CEP-1 in the presence of ^3^H-SAM, PRMT-5 did not methylate the full-length CEP-1 or its truncated fragments (Figure S3C), suggesting that PRMT-5 may act by other mechanisms to regulate CEP-1 transcriptional activity rather than directly methylates CEP-1. For example, PRMT-5 might function through other factors in complex with CEP-1 to affect its transcriptional activity.

### CBP-1 Forms Complex with CEP-1 and Can Be Methylated by PRMT-5

**Figure 5:**
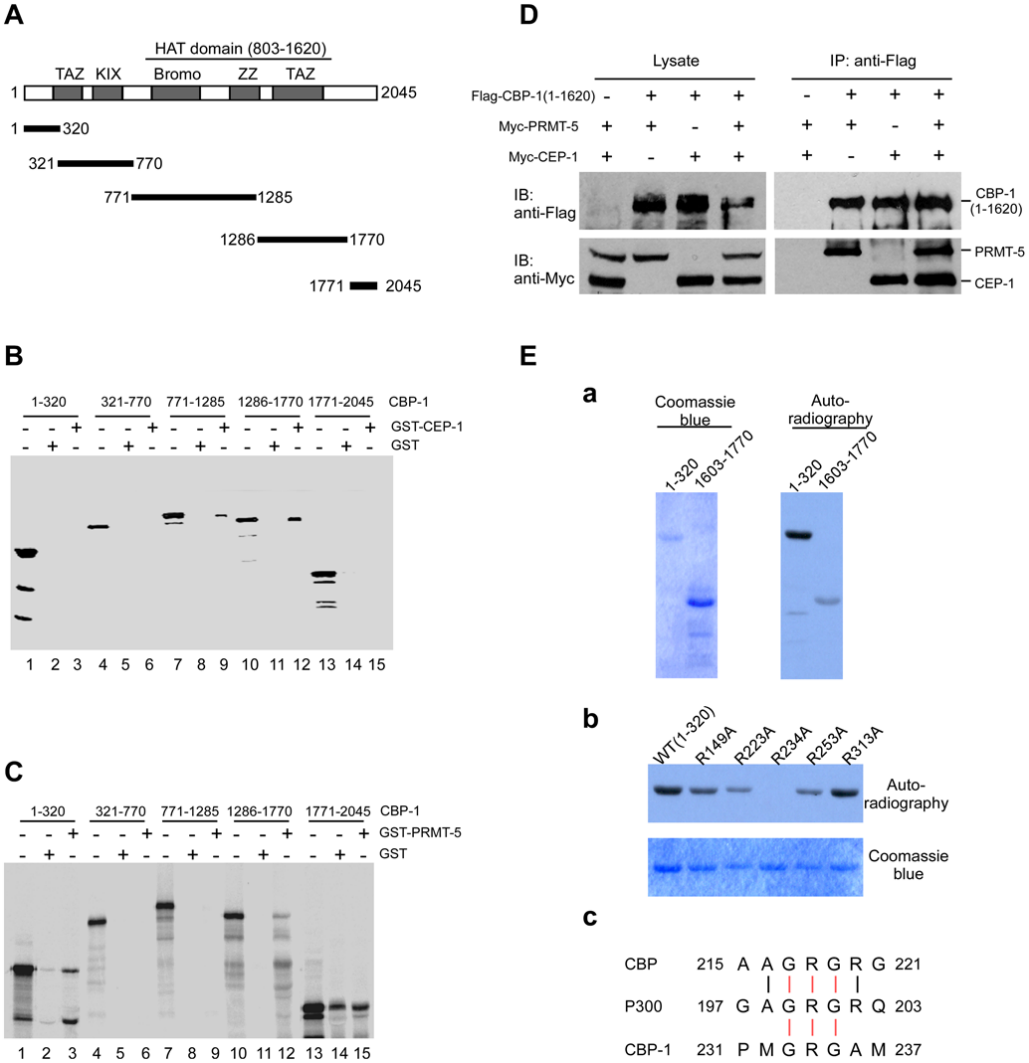
CBP-1 forms complex with CEP-1 and PRMT-5 and can be methylated by PRMT-5. (A) The schematic representation of CBP-1 full-length protein and truncation mutants. Specific motifs and domains are noted and shown in grey. (B) CBP-1 interacts with CEP-1 *in vitro*. GST pull-down assay was performed as in Figure 4E. In lanes l, 4, 7, 10 and 13, 10% of the *in vitro* translated proteins used for interaction were loaded. (C) PRMT-5 interacts with CBP-1 *in vitro*. Interactions between GST-RMT-5 and truncated CBP-1 proteins as indicated in (A) were detected as in (B). (D) PRMT-5, CBP-1 and CEP-1 form complex in mammalian cells. Flag-CBP-1(1–1620), Myc-PRMT-5 and Myc-CEP-1 were co-expressed in combinations as indicated in HEK293 cells. Immunoprecipitations (IP) were performed using anti-Flag antibody and precipitated proteins were resolved on SDS-PAGE and detected by immuno-blotting (IB) with indicated antibodies. (E) CBP-1 is methylated by PRMT-5. (a) Recombinant CBP-1(1–320) and CBP-1(1603–1770) were incubated with PRMT-5 and ^3^H-SAM for 1 h and resolved on SDS-PAGE (left panel). The methylation signals were detected by autoradiography (right panel). (b) R234A mutation abrogates the methylation of CBP-1(1–320) by PRMT-5. Wild-type and mutant CBP-1(1–320) proteins were tested for methylation as in (a). The top panel indicates the methylation of wild-type and mutant CBP-1(1–320) proteins, and the bottom panel indicates the coomassie-blue staining of these proteins. (c) The conserved arginine methylation sites in human CBP, p300 and *C. elegans* CBP-1 are shown.

**Figure 6:**
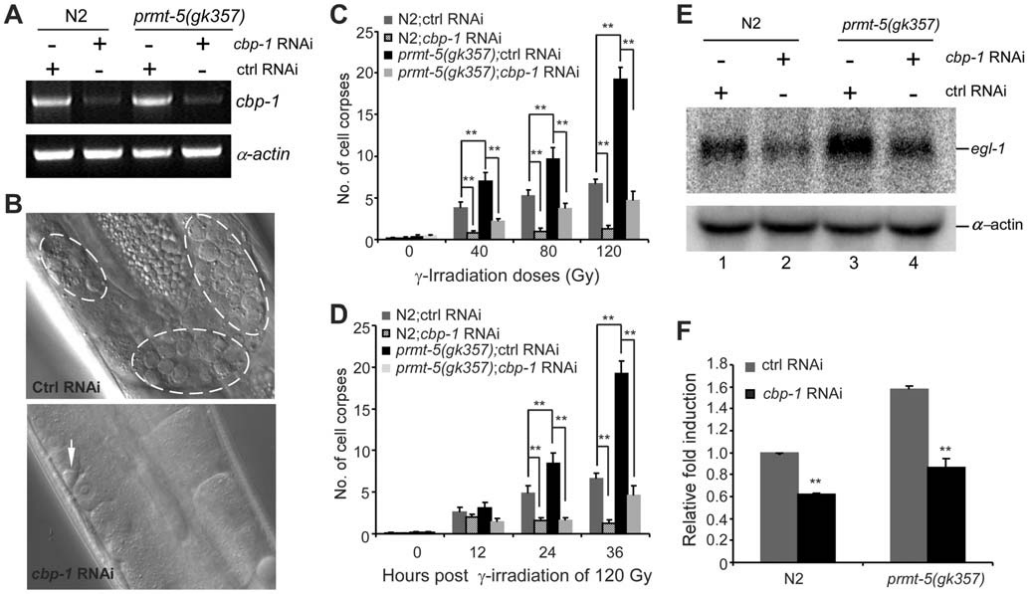
*cbp-1* RNAi inhibits DNA damage-induced excessive apoptosis in *prmt-5(gk357)* mutants. (A) *cbp-1* RNAi performed in L4-stage animals decreases the mRNA level of *cbp-1*. Indicated animals at L4 stage were treated with *cbp-1* RNAi. RNA was prepared 36 h later and *cbp-1* expression level was detected by using semi-quantitative RT-PCR. Actin mRNA was used as internal control. (B) *cbp-1* RNAi performed in L4-stage animals suppresses the excessive germ cell apoptosis induced by γ-irradiation in *prmt-5(gk357)* animals. Representative images of germ cell corpses 36 h post irradiation of 120 Gy in control RNAi- and *cbp-1* RNAi-treated *prmt-5(gk357)* worms are shown. Germ cell corpses are indicated with fragmented circles (top panel) and an arrow (bottom panel). (C, D) Quantitative analyses of *cbp-1* RNAi on DNA damage-induced germ cell apoptosis in N2 and *prmt-5(gk357)* worms. *cbp-1* RNAi was performed as above. Indicated animals were treated with different doses of γ-irradiation and germ cell corpses were scored 36 h post irradiation (C), or animals were irradiated with γ-ray of 120 Gy and germ cell corpses were scored at different time points post irradiation (D). Error bars represents SEM. Indicated comparisons were performed by using unpaired *t*-test. Double asterisks indicate p<0.001. (E) *cbp-1* RNAi suppresses *egl-1* expression induced by γ-irradiation. *cbp-1* RNAi was performed in L4-stage animals. Indicated worms at young adult stage were irradiated with γ-ray of 120 Gy and total RNA were prepared 24 h post irradiation. *egl-1* expression was detected by using *egl-1* cDNA as probe. α-actin mRNA was probed as loading control of samples. Three independent Northern blot analyses were performed and the representative images are shown. (F) Relative fold induction of *egl-1* mRNA by γ-irradiation in *cbp-1* RNAi-treated animals. *egl-1* fold induction was averaged from three independent Northern blot analyses, which was quantified by using the software ImageQuant 5.2 and normalized with α-actin mRNA. Error bars represent SEM. Comparisons were made between control RNAi and *cbp-1* RNAi treatment. Double asterisks indicate p<0.001.

To determine whether PRMT-5 acts through other CEP-1 cofactors, we sought to identify proteins that likely function together with CEP-1. In mammals, p300/CBP was found to act as a transcription coactivator of p53, and the acetylation of p53 by p300 plays an important role in p53 stabilization in response to DNA damage [4]–[6]. In addition, p300-mediated histone acetylation also contributes to the transcription of p53 target genes [27]. In *C. elegans*, the p300/CBP homolog CBP-1 was shown to regulate the differentiation of some embryonic cell types [28],[29], and CBP-1 may function in concert with the transcription factor LIN-1 to negatively regulate vulva cell specification [30]. However, it is not known whether CBP-1 could act together with CEP-1 to control gene expression in response to DNA damage. We explored this possibility first by checking if CEP-1 could interact with CBP-1. Using GST pull-down assay, we found that GST-CEP-1 fusion protein interacted with ^35^S-labeled CBP-1(771–1285) and CBP-1(1286–1770), which are within the HAT domain of CBP-1 (Figure 5A and 5B), indicating that CBP-1 and CEP-1 directly interact with one another. Moreover, partial inactivation of *cbp-1* by RNAi significantly suppressed DNA damage-induced apoptosis and *egl-1* expression in wild-type animals (Figure 6, see below and Materials and Methods), suggesting that CBP-1 is important for CEP-1 transcriptional activity. All together, these findings suggest that CBP-1 likely acts as a cofactor of CEP-1 in *C. elegans*.

Because both PRMT-5 and CBP-1 can interact with CEP-1, we wondered whether PRMT-5 and CBP-1 could directly interact with one another. Using GST pull-down assay, we found that GST-PRMT-5 interacted with ^35^S-labeled N-terminal fragment (1–320) and a fragment within the HAT domain (1286–1770) of CBP-1 (Figure 5C), indicating that PRMT-5 binds to at least two sites in CBP-1. These findings suggest that PRMT-5, CEP-1 and CBP-1 likely form a complex. To further prove this, we tested whether these proteins could interact in HEK293 cells by co-expressing Myc-PRMT-5, Myc-CEP-1 and Flag-CBP-1(1–1620) in different combinations and performing immunoprecipitation. As shown in Figure 5D, PRMT-5 and CEP-1 were associated with CBP-1 in HEK293 cells when they were individually co-expressed with CBP-1(1–1620). Furthermore, when these three proteins were co-expressed, both PRMT-5 and CEP-1 were pulled down by CBP-1 as well (Figure 5D), providing further evidence that these three proteins can form a complex.

We next examined whether CBP-1 could be modified by PRMT-5. When incubated with the recombinant PRMT-5 in the presence of ^3^H-SAM, the CBP-1 N-terminal fragment containing amino acids 1–320 was strongly methylated. Another fragment (amino acids 1603–1770) in the HAT domain was weakly methylated (Figure 5E(a)). Moreover, we found that a point mutation, R234A, completely abolished the N-terminal methylation of CBP-1 by PRMT-5 (Figure 5E(b)), indicating that R234 is the major residue for PRMT-5-mediated CBP-1 methylation. Importantly, the residue R234 is located in a GRG motif which is also present in the N-termini of human p300 and CBP (Figure 5E(c)), implying that mammalian PRMT5 can also modify CBP/p300 to affect their functions.

### Reduction of *cbp-1* Activity Suppresses IR-Induced Apoptosis in *prmt-5(gk357)* Animals

**Figure 7:**
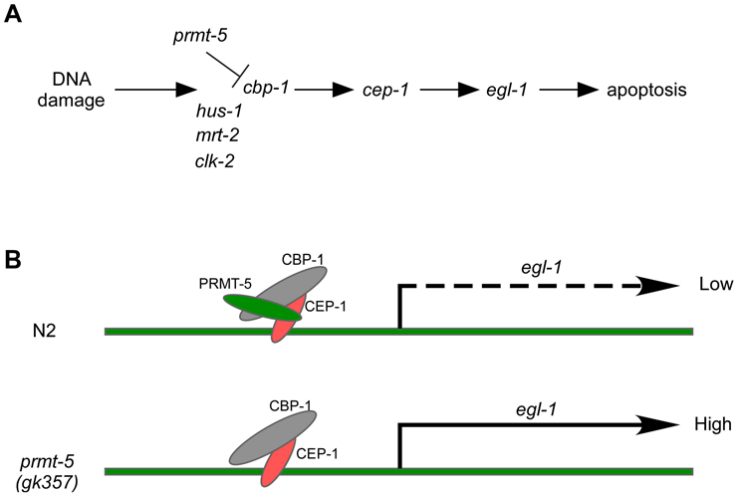
A proposed model for PRMT-5 to regulate DNA damage-induced apoptosis in *C. elegans*. (A) The genetic pathway for *prmt-5* to regulate DNA damage-induced apoptosis. (B) A proposed molecular model that PRMT-5 functions together with CEP-1 and CBP-1 to regulate *egl-1* expression in response to DNA damage.

Our biochemical data suggest that PRMT-5 likely regulates CEP-1 transcriptional activity through the CEP-1 cofactor CBP-1. To further determine this, we investigated the cellular effect of *cbp-1* on *prmt-5*-mediated apoptosis in response to DNA damage by performing *cbp-1* RNAi and examining whether it affects IR-induced apoptosis in *prmt-5(gk357)* animals. Because *cbp-1* RNAi treatment of early larvae (L1–L2 stage) gave rise to cell cycle arrest in adult germlines, we performed *cbp-1* RNAi in L4-stage animals to partially inactivate *cbp-1* (Figure 6A, Text S1, Figure S4A). Such *cbp-1* RNAi treatment did not obviously change the numbers of mitotic nuclei in germlines of wild-type, *prmt-5(gk357)* or *ced-1(e1735)* animals as compared with control RNAi (Figure S4B). In addition, *cbp-1* RNAi performed in L4-stage animals did not affect the number of germ cell corpses in *ced-1(e1735)* worms (Figure S4C). These data indicate that *cbp-1* RNAi treatment of L4-stage animals does not affect either germline development or physiological germ cell death. Thus *cbp-1* RNAi was carried out in L4-stage animals in our following experiments. Compared with control RNAi treatment, *cbp-1* RNAi caused a strong reduction of germ cell corpses in wild type when exposed to different doses of γ-irradiation (Figure 6C). Similarly, at different time points post γ-irradiation of 120 Gy, *cbp-1(RNAi)* animals also displayed significantly fewer cell corpses than control RNAi-treated wild-type animals (Figure 6D). In agreement with this, the induction of *egl-1* expression by γ-irradiation of 120 Gy in *cbp-1(RNAi)* animals was decreased by about 40% compared to that in control RNAi-treated wild-type worms (Figure 6E (lanes 1–2) and Figure 6F), suggesting that CBP-1 functions to promote CEP-1 transcriptional activity. In *prmt-5(gk357)* animals, γ-irradiation induced excessive germ cell apoptosis in a dose-dependent manner, but it was strongly suppressed by *cbp-1* RNAi (Figure 6B–6D). Consistently, the IR-induced over up-regulation of *egl-1* in *prmt-5(gk357)* animals was significantly decreased by *cbp-1* RNAi treatment (Figure 6E (lanes 3–4) and Figure 6F). These findings suggest that *cbp-1* functions downstream of *prmt-5*, providing further evidence that PRMT-5 likely acts through CBP-1 to regulate CEP-1 transcriptional activity (Figure 7).

## Discussion

Aberrant expression of PRMT5 is associated with many cancer types such as lymphoma, leukemia, gastric carcinoma and testicular tumors [26],[31],[32]. It has been shown that overexpression of PRMT5 in lymphoma is correlated to a global increase of H4R3 and H3R8 symmetric dimethylation, which likely suppresses the expression of the tumor suppressor gene ST7 to affect tumorigenesis [26]. However, whether and how PRMT5 controls the expression of apoptosis-related genes to affect cell proliferation, apoptosis, as well as tumorigenesis remains poorly understood. Here we have shown that the *C. elegans* arginine methyltransferase PRMT-5 plays an important role in DNA damage-induced apoptosis. *prmt-5* loss-of-function mutation does not affect developmental cell deaths but leads to excessive germ cell apoptosis in response to DNA damage, suggesting that *prmt-5* negatively regulates DNA damage-induced apoptosis in *C. elegans*. Our further genetic analyses indicate that mutations of *cep-1*/p53 and genes in the core cell death pathway suppress IR-induced excessive germ cell apoptosis in *prmt-5(gk357)* mutants, indicating that *prmt-5*-mediated apoptosis following DNA damage is dependent on *cep-1*/p53 and requires the core cell death pathway (Figure 7A). Meanwhile, we found that mutations in checkpoint genes significantly inhibited IR-induced excessive germ cell death in *prmt-5(gk357)* mutants, suggesting that checkpoint signaling pathways are important for *prmt-5*-mediated apoptosis (Figure 7A). Importantly, loss of *prmt-5* function causes a specific over up-regulation of the cell death initiator EGL-1 in response to DNA damage, which is directly responsible for the excessive germ cell apoptosis observed in *prmt-5(gk357)* mutants. Collectively, these findings demonstrate that PRMT-5 is a negative regulator of CEP-1/p53-dependent cell death pathway in *C. elegans*.

Our results have suggested a novel mechanism underlying *cep-1*/p53-dependent cellular response to DNA damage in *C. elegans*. In *C. elegans* germ cells, it seems that PRMT-5 does not affect the symmetric dimethylation of H4R3 although mammalian and plant PRMT5 has been reported to do so [26],[33]. Presently we can not exclude the possibility that PRMT-5 methylates other arginine residues on histone H4 to regulate CEP-1/p53-dependent gene expression, but it seems less likely that PRMT-5 modifies H3R8 to affect transcription in *C. elegans* since PRMT-5 does not methylate histone H3 *in vitro* (Figure S3A). Regardless of histone arginine methylation, our results have revealed a novel regulatory mechanism underlying DNA damage-induced *egl-1* expression. The fact that PRMT-5 interacts with but does not methylate CEP-1 implies that PRMT-5 likely regulates CEP-1 transcriptional activity by affecting a CEP-1 cofactor(s). In support of this, we have identified CBP-1 as a cofactor of CEP-1/p53, which is supported by two lines of evidence: firstly, CBP-1 and CEP-1 physically interact with one another both *in vitro* and in mammalian cells; secondly, reducing *cbp-1* expression significantly suppressed *cep-1*-dependent germ cell apoptosis and *egl-1* transcription in response to DNA damage. Moreover, we found that PRMT-5 can form complex with CEP-1 and CBP-1, and PRMT-5 can methylate the residue R234 in the N-terminus of CBP-1. Therefore PRMT-5 likely affects CEP-1 transcriptional activity through its effect on CBP-1 despite that it may not be the sole target of PRMT-5. In agreement with this notion, we found that partial inactivation of *cbp-1* by RNAi significantly suppressed the excessive germ cell apoptosis in *prmt-5(gk357)* animals following DNA damage. Consistently, *cbp-1* RNAi significantly reduced *egl-1* expression in *prmt-5(gk357)* mutants. Based on these experimental findings, we propose a possible model for PRMT-5 functioning through CBP-1 and CEP-1 to negatively regulate DNA damage-induced apoptosis in *C. elegans*: In wild-type animals, PRMT-5 and CBP-1 are likely recruited by CEP-1 to form a complex so that CBP-1 is methylated by PRMT-5, which represses the capacity of CBP-1 for enhancing CEP-1-dependent transcription of *egl-1*. In this case, *egl-1* expression is maintained at a proper level to avoid excessive germ cell apoptosis after DNA damage (Figure 7B). In *prmt-5(gk357)* animals, the repression on CBP-1 by PRMT-5 is removed, thus CBP-1 promotes the transcriptional activity of CEP-1 by mechanisms yet to be uncovered, leading to high expression level of *egl-1* following DNA damage, which in turn causes excessive germ cell apoptosis (Figure 7B).

Although *ced-13* was reported to be a transcription target of CEP-1, we found that IR-induced *ced-13* expression is indistinguishable between wild type and *prmt-5(gk357)* mutants and *ced-13* loss of function does not suppress IR-induced apoptosis in *prmt-5(gk357)* animals. These results suggest that the regulatory effect of PRMT-5 on IR-induced CEP-1 transcriptional activity is likely specific to *egl-1* transcription. It was found recently that IR-induced *ced-13* expression is only detected in somatic tissues but not in germline where DNA damage-induced apoptosis takes place [24], suggesting that *ced-13* may play a role in DNA damage response in soma while *egl-1* is the major CEP-1 target responsible for initiating germ cell apoptosis after DNA damage. In addition, although PRMT-5 is implicated in maintaining genome stability [21], our results indicate PRMT-5 likely acts mainly in germline to regulate DNA damage response since the survival of *prmt-5(gk357)* embryos was comparable to that of wild-type animals after IR treatment (Table S1), which also suggests that *prmt-5* probably does not obviously affect the repair of DNA lesions caused by γ-irradiation. As *prmt-5(gk357)* worms also displayed similar cell cycle arrest in germline mitotic region to that in wild-type animals upon DNA damage (data not shown), it seems that *prmt-5* acts differently in sensing DNA damage from checkpoint genes. Thus, apart from a role in regulating DNA damage-induced germ cell apoptosis, it remains to be elucidated how *prmt-5* inactivation causes increased accumulation of mutation observed previously [21].

Because strong loss of function of *cbp-1* causes lethality [29], we could only analyze the role of *cbp-1* in *prmt-5*-mediated germ cell apoptosis in response to DNA damage by using the partial loss-of-function mutation of *cbp-1* (*cbp-1* RNAi), which suppressed IR-induced germ cell apoptosis in *prmt-5(gk357)* mutant worms to a less extent than suppressed by the strong loss of function of *cep-1*. Therefore we can not conclude that PRMT-5 acts solely through CBP-1 based on the *cbp-1* RNAi results. Nevertheless, in agreement with our findings that PRMT-5 likely regulates CEP-1 activity through CBP-1 in *C. elegans*, a recent study also identified human PRMT5 as a negative regulator of p53-mediated apoptosis involving p300/CBP in mammalian cells. Jansson et al. reported that PRMT5 is associated with the CBP-binding protein Strap. As a result, PRMT5 is recruited to p53 to methylate the latter on the arginine residues in an RGRER motif [34]. Similar to that in *C. elegans*, inactivation of PRMT5 by siRNA significantly enhanced DNA damage-induced apoptosis of mammalian cells [34]. Unlike human PRMT5, however, we did not find that *C. elegans* PRMT-5 modify CEP-1, which is consistent with that CEP-1 does not contain an RGRER motif as human p53 [34]. Thus our findings suggest a possibility that human PRMT5 may affect p53 activity through additional mechanisms except for p53 arginine methylation. For example, it is likely that human PRMT5 can function similarly to its *C. elegans* counterpart to regulate p300/CBP activity by arginine methylation. Previously, it has been shown that the coactivator-associated arginine methyltransferase 1 (CARM1/PRMT4) can methylate p300/CBP in the KIX domain to disable the interaction between the p300/CBP KIX domain and the kinase inducible domain (KID) of CREB, which blocks CREB-dependent transcription of genes such as Bcl-2 [16]. On the other hand, CARM1-mediated methylation of p300/CBP enhances nuclear hormone receptor (NR)-dependent gene transcription [16],[17]. These findings indicate that the arginine methylation of p300/CBP is one of the mechanisms underlying transcription regulation. In *C. elegans*, PRMT-5 methylates an arginine residue located in the GRG motif in the N-terminus of CBP-1. Interestingly, this GRG motif also exists in the N-termini of mammalian p300 and CBP. Furthermore, it has been found that PRMT5 is present in the p53 co-activator complex containing p300/CBP in mammalian cells [34]. Thus, it will be very important to determine whether human PRMT5 can methylate p300/CBP on the same arginine residue to regulate its coactivator activity in promoting apoptosis-related gene transcription. Further in-depth mechanistic studies in both *C. elegans* and mammalian cells will be needed to establish the role of the site-specific methylation of p300/CBP by PRMT5 in regulating p53-dependent apoptosis.

## Material and Methods

### *C. elegans* Strains and Genetics

*prmt-5(gk357)* and *cep-1(gk138)* deletion strains were generated by Dr. Donald Moerman (*C. elegans* Reverse Genetics Core Facility, Vancouver, B.C., Canada) and provided by *C. elegans* Genetic Center (CGC). *ced-13(tm536)* deletion strain was provided by Dr. Shohei Mitani. Worms were cultured and maintained by using standard procedures [35]. The Bristol N2 strain was used as wild type. Deletion strains were outcrossed with N2 strain for 6 times. Double mutants were constructed with standard protocol [35].

### Molecular Biology

To make RNAi constructs of *prmt* genes, the exons 1 and 2 of *prmt-1* (nucleotide +10–954), exon 3 of *prmt-2* (nucleotide +372–755), exon 8 of *prmt-3* (nucleotide +5135–5487) and exons 4 and 5 of *prmt-5* (nucleotide +805–1536) were amplified by PCR and cloned into pPD129.36, respectively. RNAi constructs for *prmt-4*, -*6* and *cbp-1* were obtained from an RNAi library (Geneservice Ltd). For bacterial and mammalian expression of PRMT-5 and CEP-1, the cDNAs of *prmt-5* and *cep-1* were cloned into the bacterial expression vectors pET21a and pGEX4-T-2 and the mammalian expression vectors pCMV-myc and pCMV-tag2B, respectively. The full-length cDNA of *cbp-1* was obtained by ligating the cDNA fragments from the following *yk* cDNA clones: *yk1426d05*, *yk838d03*, *yk822d08*, *yk1753c05* and *yk1403a01*, and verified by sequencing. Different *cbp-1* cDNA fragments were amplified from the full-length cDNA by PCR and cloned into bacterial and mammalian expression vectors as above. His6-tagged and GST-fusion proteins were expressed and purified as described previously [36].

### RNA Interference

RNA interference was performed by using the feeding assay. Briefly, The L1 larvae were grown on RNAi plates seeded with bacteria HT115(DE3) expressing dsRNA of individual *prmt* genes. The progeny maintained on the RNAi plates were synchronized to young adult stage and treated with different doses of γ-irradiation as described below. For *cbp-1* RNAi treatment, worms at early L4 stage were cultured on RNAi plates seeded with bacteria HT115(DE3) expressing dsRNA of *cbp-1*. After reaching young adult stage, *cbp-1(RNAi)* worms were irradiated with γ-ray and germ cell corpse phenotype and Northern blot assays were analyzed 36 h or 24 h post irradiation, respectively.

### Germ Cell Apoptosis Assays

Synchronized young adult animals were irradiated with γ-ray by using a ^60^Co source located in the Peking University Health Science Center. Irradiated animals were put back to culture at 20°C to different time points. Worms with normal germline morphology were scored for germ cell corpses by using Nomarski optics. For acridine orange (AO) staining of germ cell corpses, irradiated worms were incubated in M9 medium containing AO (50 µg/ml) and bacteria OP50 in dark for 2 h. Worms were recovered on NGM plates for another 2 h and examined with epifluorescence microscopy. To induce germline apoptosis with ENU, young adult animals were incubated in M9 medium containing OP50 bacteria and ENU of different concentrations (0, 1.0, 2.5 and 5.0 mM) for 4 h. Worms were then recovered on NGM plates for 24 h and germ cell corpses were scored with DIC microscopy.

### Northern Blot Analysis

Worms synchronized to young adult stage were irradiated with γ-ray of 120 Gy as described above. 24 h later, irradiated worms were harvested and total RNA was extracted using the Trizol reagent (Invitrogen). 20 µg of total RNA was denatured and resolved on 1.2% agarose-formaldehyde gel in MOPS buffer (20 mM 3-Morpholinopropam sulfonsaure, 2 mM Sodium acetate, 1 mM EDTA) and further blotted onto a nylon membrane. For hybridization, the membrane was incubated with ^32^P-labeled probes prepared from *egl-1* cDNA in a buffer containing 7% SDS, 1% BSA, 1 mM EDTA, 250 mM Na_n_PO_4_ (pH7.2). The membrane was extensively washed and exposed to a phosphor imager screen (Amersham). To examine the equal loading of total RNA samples, the membrane was striped and re-probed with ^32^P-labeled probes of α-actin. Relative fold induction of *egl-1* mRNA was quantified with the ImageQuant 5.2 software and normalized with α-actin mRNA.

### Germline Expression of GFP::PRMT-5

For expression of GFP::PRMT-5 fusion protein in germline, we cloned the cDNA of *prmt-5* into the germline expression vector pTE5 in frame with GFP (P*_pie-1_ gfp::prmt-5*). This PRMT-5-expression vector was linearized with SacII and co-injected at the concentration of 1 µg/ml into worm germline with the SacI-digested N2 genomic DNA (60 µg/ml) and the SalI-linearized injection marker pTG96 (1 µg/ml) which expresses P*_sur-5_gfp* only in somatic tissues. The transgenic extrachromasomal arrays from F2 and F3 generations were integrated into worm genome by γ-irradiation. Gonads of integrated transgenic worms were dissected out and stained with Hoechst 33342 (4 µM) to examine the localization of GFP::PRMT-5 with epifluorescence scope, and the germline expression of GFP::PRMT-5 was further confirmed by using Western blot assay.

### Mammalian Cell Culture, Transfection, and Immunoprecipitation

Human embryonic kidney cells (HEK293) grown in Dulbecco's modified Eagle's medium (HyClone) supplemented with 10% fetal bovine serum (HyClone) were transfected with 2.0 µg of mammalian vectors expressing worm proteins with different tags (i.e., pCMV-myc-*prmt-5*, pCMV-tag2B-*cep-1*, pCMV-myc-*cep-1*, pCMV-tag2B-*cbp-1(1–1620)*) by using the calcium phosphate-mediated transfection assay. 36 h after transfection, cells were harvested and lysed in a buffer containing 50 mM Tris (pH 8.0), 150 mM NaCl, 0.5% sodium deoxycholate, 1% Triton X-100, 1 mM phenylmethylsulfonyl fluoride (PMSF). The lysate was incubated with anti-Flag antibody (M2)-conjugated agarose beads (Sigma) for more than 2 h at 4°C. The beads were washed extensively in a buffer containing 50 mM Tris (pH 8.0), 150 mM NaCl, 1 mM PMSF and 1% NP-40 and bound proteins were eluted with protein sample buffer. The eluted proteins were resolved on SDS-PAGE and detected with Western blot assay.

### GST Pull-Down Assay

For GST pull-down assay, purified GST or GST fusion proteins were immobilized on glutathione-Sepharose beads and incubated with [^35^S]methionine-labeled proteins at 4°C for more than 2 h. The beads were washed extensively and bound proteins were eluted and separated on 12% SDS-PAGE and exposed to X-ray film or phosphor imager screen (Amersham) for autoradiography.

### *In Vitro* Protein Methylation

PRMT-5His6 or GST-PRMT-5 was respectively incubated with proteins including core histones, Myelin basic protein (MBP), full-length and truncated GST-CEP-1 proteins, wild-type and mutant CBP-1(1–320)His6 and CBP-1(1603–1770)His6 in the presence of 0.55 µCi of ^3^H-*S*-AdoMet in PRMT assay buffer (25 mM Tris, pH 7.5, 1 mM EDTA, 1 mM EGTA, and 1 mM PMSF) for 1 h at 30°C in a final volume of 20 µl. Reactions were stopped by adding SDS sample buffer and heated at 100°C for 10 min. Samples were resolved on 12% SDS-PAGE and further stained with Coomassie blue and dried to expose to X-ray film for autoradiography.

## Supporting Information

Figure S1 Characterization of *prmt* genes and DNA damage-induced apoptosis in worms treated with RNAi of *prmt* genes. (A) Phylogenetic analysis of human PRMTs and *C. elegans* PRMTs. Protein sequences are aligned by using Clustal W and phylogenetic tree is built by using the software MEGA 4.1. (B) Comparison of human PRMT1 and *C. elegans* PRMT proteins. Specific motifs for protein arginine methyltransferase are: Motif I (VLD/EVGxGxG), Post I (V/IxG/AxD/E), Motif II (F/I/VDI/L/K), Motif III (LR/KxxG), and THW loop [4]. x represents any amino acid residue. Motif I, Post I and the THW loop form part of the AdoMet-binding pocket [5]. (C) Quantification of germ cell apoptosis induced by γ-irradiation in worms pre-treated with RNAi of *prmt* genes. Young adult N2 worms grown on RNAi plates were irradiated with γ-ray of 120 Gy and germ cell corpses from one gonad arm were scored 36 h post irradiation. At least 20 animals were scored. Error bars represent SEM. Comparisons were performed between control RNAi and individual *prmt* gene RNAi with unpaired t-test. Double asterisks indicate p<0.001.
(0.13 MB TIF)

Figure S2 Amino acid sequence alignment of human PRMT5 (H.s.PRMT5), yeast Skb1 (S.p.SKB1) and *C. elegans* PRMT-5 (C.e.PRMT-5). Identical residues are shaded in black and similar residues are shaded in gray. Characteristic motifs for protein arginine methyltransferase are boxed and indicated.
(0.38 MB TIF)

Figure S3 *prmt-5(gk357)* does not affect *in vivo* H4R3 symmetric dimethylation and PRMT-5 does not methylate CEP-1. (A) PRMT-5 methylates histone H4 but not H3 *in vitro*. Core histones and myelin basic protein (MBP) were incubated with PRMT-5 and ^3^H-SAM for 1 h and resolved on SDS-PAGE (left panel). Methylation signals were detected with autoradiography (right panel). (B) *prmt-5(gk357)* does not affect histone H4 symmetric dimethylation *in vivo*. Gonads from N2 and *prmt-5(gk357)* were stained with anti-H4R3sMe2 antibody (shown in Red) and germ cells at pachytene stage are shown. Nuclei are stained with DAPI (4′,6-diamidino-2-phenylindole) (shown in Green). (C) PRMT-5 does not methylate CEP-1 *in vitro*. Core histones, MBP, GST, GST-CEP-1 proteins as indicated were incubated with PRMT-5 and ^3^H-SAM for 1 h, respectively, and resolved on SDS-PAGE. Methylation signals were detected by autoradiography. Histone H4 and MBP were indicated by arrows.
(3.61 MB TIF)

Figure S4 *cbp-1* RNAi performed in L4-stage animals does not affect germline proliferation and physiological germ cell death. (A) *cbp-1* RNAi performed in L4-stage animals decreases CBP-1 protein expression. Wild-type (N2) animals at L4 stage were treated with *cbp-1* RNAi. 36 h later, CBP-1 expression in germline was detected by immunostaining using anti-CBP-1 antibody. Nuclei were stained by DAPI. Images of CBP-1 (red), nuclei (blue) and merged images of oocyte regions are shown for control RNAi- and *cbp-1* RNAi-treated worms. Arrows indicate the nuclear localization of CBP-1. (B) *cbp-1* RNAi performed in L4-stage animals does not affect germline proliferation. Indicated animals at L4 stage were treated with *cbp-1* RNAi. 36 h later, germlines were stained with DAPI and nuclei in germline mitotic regions were counted. Error bars represent SEM. (C) *cbp-1* RNAi performed in L4-stage animals does not affect physiological germ cell death. *ced-1(e1735)* animals at L4 stage were treated with *cbp-1* RNAi, 36 h later, germ cell corpses were scored and analyzed as described previously. Error bars represent SEM.
(4.08 MB TIF)

Table S1 *prmt-5(gk357)* does not affect the survival of progeny following exposure to γ-irradiation.
(0.03 MB DOC)

Text S1 Supporting material and methods.
(0.03 MB DOC)
