# Supplementary figures and images for: Caenorhabditis elegans Protein Arginine Methyltransferase PRMT-5 Negatively Regulates DNA Damage-Induced Apoptosis
Source: PLoS Genet. 2009 Jun 12;5(6):e1000514. doi: 10.1371/journal.pgen.1000514 (PMC2691592; doi:10.1371/journal.pgen.1000514)

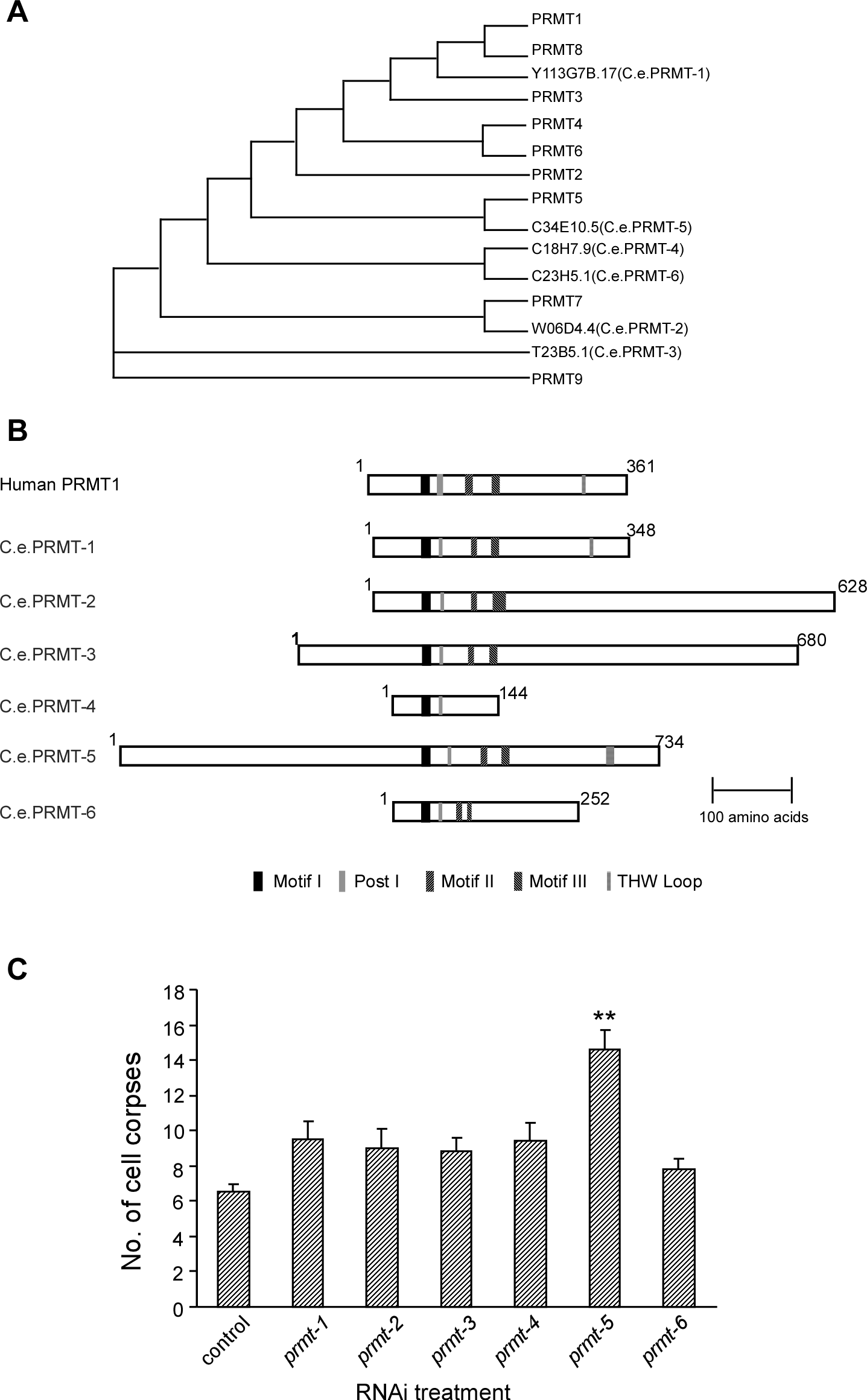

Supplement: Figure S1 — Characterization of prmt genes and DNA damage-induced apoptosis in worms treated with RNAi of prmt genes. (A) Phylogenetic analysis of human PRMTs and C. elegans PRMTs. Protein sequences are aligned by using Clustal W and phylogenetic tree is built by using the software MEGA 4.1. (B) Comparison of human PRMT1 and C. elegans PRMT proteins. Specific motifs for protein arginine methyltransferase are: Motif I (VLD/EVGxGxG), Post I (V/IxG/AxD/E), Motif II (F/I/VDI/L/K), Motif III (LR/KxxG), and THW loop [4]. x represents any amino acid residue. Motif I, Post I and the THW loop form part of the AdoMet-binding pocket [5]. (C) Quantification of germ cell apoptosis induced by γ-irradiation in worms pre-treated with RNAi of prmt genes. Young adult N2 worms grown on RNAi plates were irradiated with γ-ray of 120 Gy and germ cell corpses from one gonad arm were scored 36 h post irradiation. At least 20 animals were scored. Error bars represent SEM. Comparisons were performed between control RNAi and individual prmt gene RNAi with unpaired t-test. Double asterisks indicate p<0.001. (0.13 MB TIF) [file pgen.1000514.s001.tif]

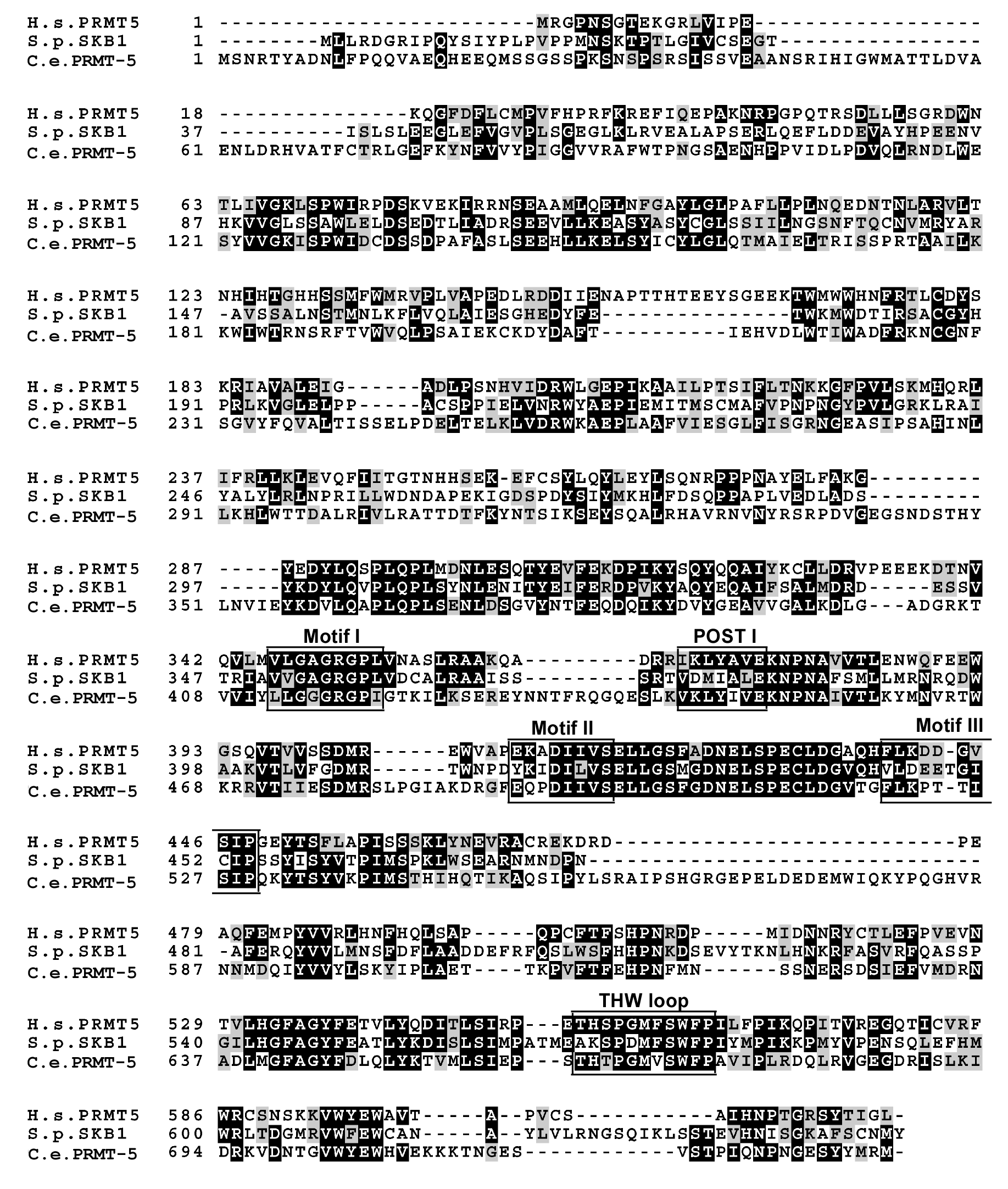

Supplement: Figure S2 — Amino acid sequence alignment of human PRMT5 (H.s.PRMT5), yeast Skb1 (S.p.SKB1) and C. elegans PRMT-5 (C.e.PRMT-5). Identical residues are shaded in black and similar residues are shaded in gray. Characteristic motifs for protein arginine methyltransferase are boxed and indicated. (0.38 MB TIF) [file pgen.1000514.s002.tif]

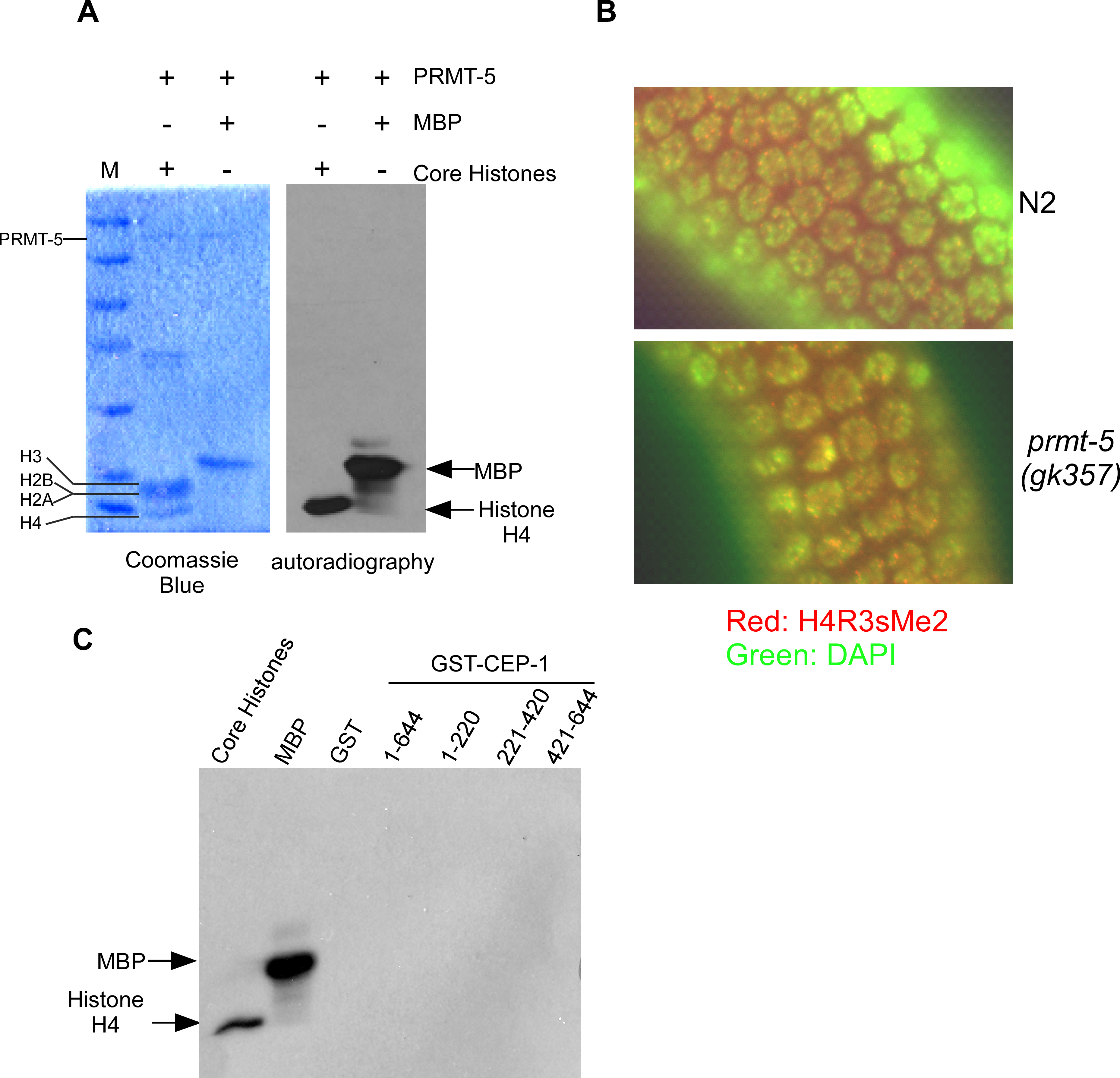

Supplement: Figure S3 — prmt-5(gk357) does not affect in vivo H4R3 symmetric dimethylation and PRMT-5 does not methylate CEP-1. (A) PRMT-5 methylates histone H4 but not H3 in vitro. Core histones and myelin basic protein (MBP) were incubated with PRMT-5 and 3H-SAM for 1 h and resolved on SDS-PAGE (left panel). Methylation signals were detected with autoradiography (right panel). (B) prmt-5(gk357) does not affect histone H4 symmetric dimethylation in vivo. Gonads from N2 and prmt-5(gk357) were stained with anti-H4R3sMe2 antibody (shown in Red) and germ cells at pachytene stage are shown. Nuclei are stained with DAPI (4′,6-diamidino-2-phenylindole) (shown in Green). (C) PRMT-5 does not methylate CEP-1 in vitro. Core histones, MBP, GST, GST-CEP-1 proteins as indicated were incubated with PRMT-5 and 3H-SAM for 1 h, respectively, and resolved on SDS-PAGE. Methylation signals were detected by autoradiography. Histone H4 and MBP were indicated by arrows. (3.61 MB TIF) [file pgen.1000514.s003.tif]

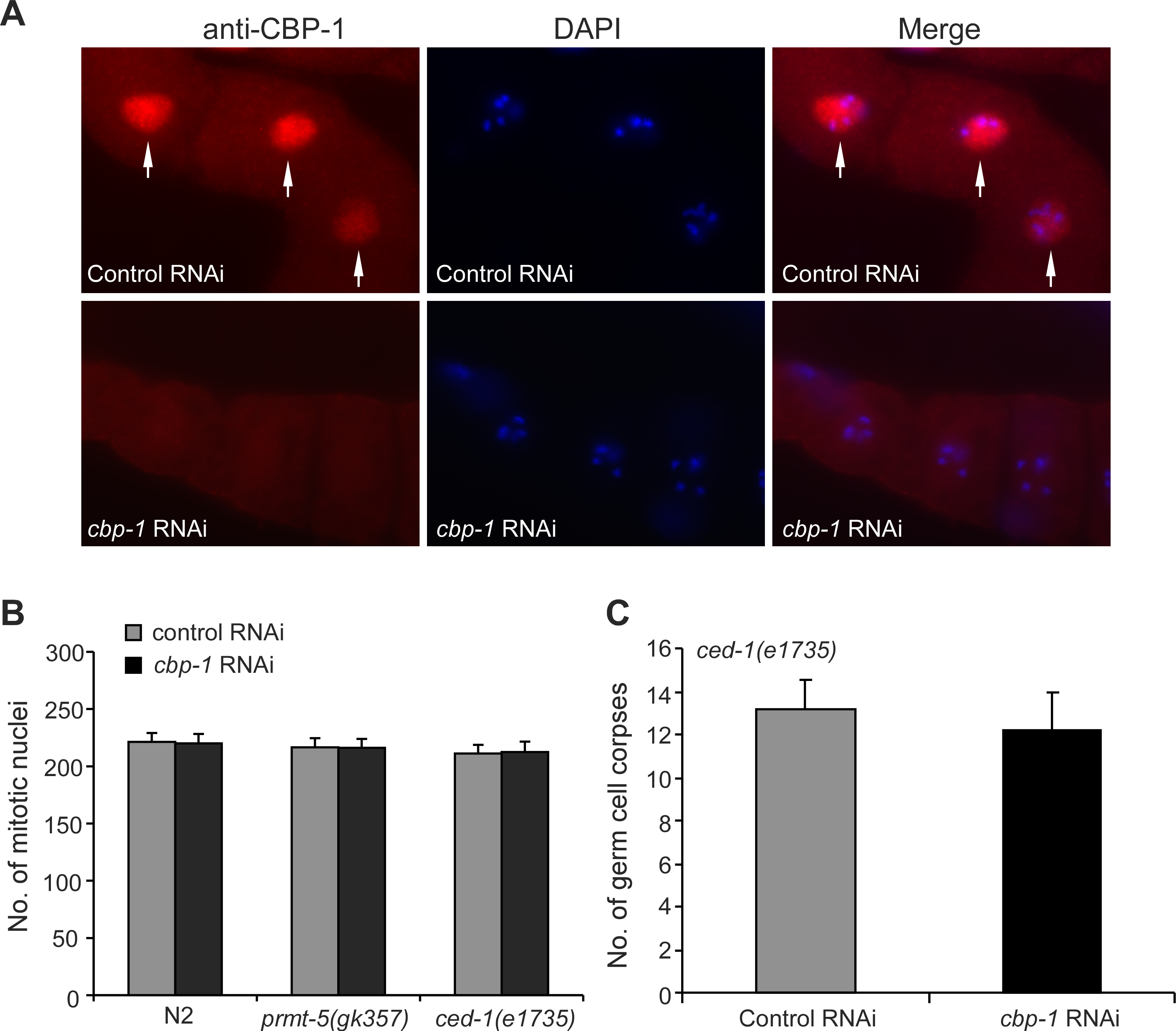

Supplement: Figure S4 — cbp-1 RNAi performed in L4-stage animals does not affect germline proliferation and physiological germ cell death. (A) cbp-1 RNAi performed in L4-stage animals decreases CBP-1 protein expression. Wild-type (N2) animals at L4 stage were treated with cbp-1 RNAi. 36 h later, CBP-1 expression in germline was detected by immunostaining using anti-CBP-1 antibody. Nuclei were stained by DAPI. Images of CBP-1 (red), nuclei (blue) and merged images of oocyte regions are shown for control RNAi- and cbp-1 RNAi-treated worms. Arrows indicate the nuclear localization of CBP-1. (B) cbp-1 RNAi performed in L4-stage animals does not affect germline proliferation. Indicated animals at L4 stage were treated with cbp-1 RNAi. 36 h later, germlines were stained with DAPI and nuclei in germline mitotic regions were counted. Error bars represent SEM. (C) cbp-1 RNAi performed in L4-stage animals does not affect physiological germ cell death. ced-1(e1735) animals at L4 stage were treated with cbp-1 RNAi, 36 h later, germ cell corpses were scored and analyzed as described previously. Error bars represent SEM. (4.08 MB TIF) [file pgen.1000514.s004.tif]
