# Supplementary material for: Caenorhabditis elegans Protein Arginine Methyltransferase PRMT-5 Negatively Regulates DNA Damage-Induced Apoptosis
Source: PLoS Genet. 2009 Jun 12;5(6):e1000514. doi: 10.1371/journal.pgen.1000514 (PMC2691592; doi:10.1371/journal.pgen.1000514)
Supplement: Table S1 — prmt-5(gk357) does not affect the survival of progeny following exposure to γ-irradiation. (0.03 MB DOC) [file pgen.1000514.s005.doc]

**Table S1.** *prmt-5(gk357)* does not affect the survival of progeny following exposure to γ-irradiation.

Irradiation Egg Laying/h/animal Survival (%)

dose (Gy) N2 *prmt-5(gk357)* N2 *prmt-5(gk357)*

0 5.8±0.6 4.6±1.1 100 98.7±0.6

40 4.5±0.7 2.2±0.4 86.8±5.2 87.7±5.6

80 3.2±0.5 1.6±0.4 75.9±9.9 75.7±5.6

120 3.3±0.8 1.0±0.3 50.5±9.2 55.4±3.1

L4-stage worms were irradiated with indicated doses. Eggs laid by irradiated worms between 24 h and 34 h after irradiation were counted for egg-laying rate. Animals hatched from these eggs and unhatched eggs were counted for the following 2 days to assess progeny survival. Numbers represent mean ± SEM.
