# Supplementary material for: Caenorhabditis elegans Protein Arginine Methyltransferase PRMT-5 Negatively Regulates DNA Damage-Induced Apoptosis
Source: PLoS Genet. 2009 Jun 12;5(6):e1000514. doi: 10.1371/journal.pgen.1000514 (PMC2691592; doi:10.1371/journal.pgen.1000514)
Supplement: Text S1 — Supporting material and methods. (0.03 MB DOC) [file pgen.1000514.s006.doc]

**Supporting Materials and Methods**

**Radiation Sensitivity Assay**

We assessed the sensitivity of *prmt-5(gk357)* mutants to γ-irradiation by using a protocol as described previously [1-3]. Briefly, worms were synchronized to L4 stage and γ-irradiated at the doses of 0, 40, 80 and 120 Gy, respectively. 24 h later, irradiated worms were transferred to fresh NGM plates. Eggs laid between 24 h and 34 h post irradiation were counted for egg-laying. Animals hatched from these eggs and unhatched eggs were counted for the following 2 days.

**Antibodies**

Anti-PRMT-5 and anti-CBP-1 antibodies were generated in rabbits by using purified recombinant PRMT-5His6 and CBP-1(1-320)His6. These antibodies were further purified for Western blot and immunostaining.

**Immunostaining**

Gonads of adult worms were dissected out and fixed with 100% methanol for 20 seconds and air-dried. Samples were then incubated in antibody buffer (PBS, 0.5% Triton X-100 and 1 mM EDTA, pH 8.0) containing 1% BSA for 2 h at room temperature to block potential non-specific binding sites of antibodies. Subsequently, the anti-H4R3sMe2 antibody (ab5823, Abcam) with a dilution of 1:2000 was incubated with gonad samples at 4 ºC overnight. After being washed with the same antibody buffer as above for 3 times, gonad samples were further incubated with goat anti-rabbit IgG-Cy3 antibody at 1:3000 dilution for 1 h. The samples were washed again for another 3 times as above and mounted to slides with the addition of DAPI (0.1 μg/ml). Fluorescent images were captured by using a Zeiss Axioimager M1 microscope. Germline CBP-1 staining was essentially performed as above by using anti-CBP-1 antibody.

**References**

1. Quevedo C, Kaplan DR, Derry WB (2007) AKT-1 regulates DNA-damage-induced germline

apoptosis in *C. elegans*. Curr Biol 17: 286-292.

2. Schumacher B, Hanazawa M, Lee MH, Nayak S, Volkmann K, et al. (2005) Translational repression of *C. elegans* p53 by GLD-1 regulates DNA damage-induced apoptosis. Cell 120: 357-368.

3. Gartner A, Milstein S, Ahmed S, Hodgkin J, Hengartner MO (2000) A conserved checkpoint pathway mediates DNA damage--induced apoptosis and cell cycle arrest in *C. elegans*. Mol Cell 5: 435-443.
